# New Charge Transfer Complexes of K^+^-Channel-Blocker Drug (Amifampridine; AMFP) for Sensitive Detection; Solution Investigations and DFT Studies

**DOI:** 10.3390/molecules26196037

**Published:** 2021-10-05

**Authors:** Reem M. Alghanmi, Maram T. Basha, Saied M. Soliman, Razan K. Alsaeedi

**Affiliations:** 1 Department of Chemistry, College of Science, University of Jeddah, P.O. 80327, Jeddah 21589, Saudi Arabia; mtbasha@uj.edu.sa (M.T.B.); razaann.m@gmail.com (R.K.A.); 2 Department of Chemistry, Faculty of Science, Alexandria University, P.O. 426, Ibrahimia, Alexandria 21525, Egypt; saied1soliman@yahoo.com

**Keywords:** amifampridine, DDQ, TCNE, charge transfer complex, spectroscopy, DFT, TD-DFT

## Abstract

UV–Vis spectroscopy was used to investigate two new charge transfer (CT) complexes formed between the K^+^-channel-blocker amifampridine (AMFP) drug and the two π-acceptors 2,3-dichloro-5,6-dicyano-*p*-benzoquinone (DDQ) and tetracyanoethylene (TCNE) in different solvents. The molecular composition of the new CT complexes was estimated using the continuous variations method and found to be 1:1 for both complexes. The formed CT complexes’ electronic spectra data were further employed for calculating the formation constants (*K_CT_*), molar extinction coefficients (*ε_CT_*), and physical parameters at various temperatures, and the results demonstrated the high stability of both complexes. In addition, sensitive spectrophotometric methods for quantifying AMFP in its pure form were proposed and statistically validated. Furthermore, DFT calculations were used to predict the molecular structures of AMFP–DDQ and AMFP–TCNE complexes in CHCl_3_. TD-DFT calculations were also used to predict the electronic spectra of both complexes. A CT-based transition band (exp. 399 and 417 nm) for the AMFP–TCNE complex was calculated at 411.5 nm (f = 0.105, HOMO-1 → LUMO). The two absorption bands at 459 nm (calc. 426.9 nm, f = 0.054) and 584 nm (calc. 628.1 nm, f = 0.111) of the AMFP–DDQ complex were theoretically assigned to HOMO-1 → LUMO and HOMO → LUMO excitations, respectively.

## 1. Introduction

The charge transfer (CT) formation includes the interaction of two or more molecular fragments by electrostatic attraction as a result of the partial transfer of charge from an e-donor to an e-acceptor. This attraction is not a stable chemical bonding, and it is weaker than covalent bonding. The CT interaction is more accurately described as a weak resonance [1,2]. However, the interaction between e-donor and e-acceptor molecules is explained using the higher occupied molecular orbital (HOMO) and lowest unoccupied molecular orbital (LUMO) interactions. The HOMO energy level of the e-donor molecule interacts with the low-lying LUMO energy level of the e-acceptor molecule to form a relatively stable intermediate complex with a small bandgap [3]. The first CT complexes were the aromatic hydrocarbon–picric acid complexes isolated by Fritzsche [4]. CT complexes are generally identified by forming intensely colored complexes that absorb visible radiation on a regular basis. They have been referred to by various names, including CT complexes, molecular complexes, addition complexes, and electron donor–acceptor complexes. CT complexes have attracted researchers’ interest because they can exhibit novel optoelectronic properties not found in the initial components, i.e., the donor and acceptor. In recent years, the investigation of new CT complexes has taken up a considerable area in chemical and biochemical research [5,6,7,8,9,10,11]. The study of CT complexation recorded several milestones in biological activity studies [9,12], DNA binding studies [13,14], and surface chemistry [14]. They are also used in a variety of applications, such as organic semiconductors [15], nonlinear optical materials [16], solar energy storage [17], and drug analysis [18].

Density functional theory (DFT) is a standard ground–state electronic structure calculation in quantum chemistry and materials science [19,20,21,22]. DFT has a wide range of applications, including providing complete and precise structural features of CT, hydrogen-bonded, and coordination complexes [23,24]. The CT complexation was recently investigated using DFT calculations to describe the donor and acceptor molecules in the CT complex [25,26]. Furthermore, the time-dependent density functional theory (TD-DFT) was applied to compute the electronic absorption spectra of the studied CT complexes [14,27].

Amifampridine (AMFP), also known as 3,4-diaminopyridine, is a K^+^-channel-blocker with limited central nervous system toxicity that has been shown to improve neuromuscular transmission [28]. The free base form of AMFP has been used to treat congenital myasthenic syndromes and Lambert–Eaton myasthenic syndrome (LEMS) since the 1990s [29]. Then, in 2006, AMFP was recommended as the first-line treatment for LEMS with ad hoc forms of the drug used because there was no commercial form. Several HPLC methods for determining the AMFP drug and its impurity in plasma were described in the literature [30,31,32,33]. However, these methods seem to be highly cost intensive. We previously investigated the synthesis and spectroscopic properties of AMFP as e-donors with various acceptors to fully understand the nature of its CT interaction [34]. We report, in this work, the formation of new CT complexes between AMFP with 2,3-dichloro-5,6-dicyano-*p*-benzoquinone (DDQ) and tetracyanoethylene (TCNE) as π-acceptors in different solvents in order to realize the drug-receptor mechanism and to develop precise and cost-effective methods for determining AMFP quantitatively. For this task, spectroscopic studies for the CT complexation between AMFP and DDQ or TCNE in various solvent systems, combined with theoretical calculations using DFT and TD–DFT methods, were performed.

## 2. Results and Discussion

### 2.1. Selection of Solvent

The reaction between AMFP and DDQ was tested in several solvents to determine the best medium for CT complex formation. We found that no single solvent was suitable for completing the CT reaction. As a result, mixed solvents systems such as acetonitrile–ethanol (ANEt), acetonitrile–dichloromethane (ANDCM), acetonitrile–dichloroethane (ANDCE), and acetonitrile–chloroform (ANCHCl_3_) were used. Throughout the study, the ideal solvent system for the CT reaction was a binary mixture of (50% AN + 50% CHCl_3_ (*v*/*v*)) and the binary mixture of (50% AN + 50% DCM (*v*/*v*)). Both systems have excellent solvating power for the reactants and yield high absorbance and high *ε_CT_* values. Several solvents, including CHCl_3_, DCM, EtOH, and MeOH, were tested for the reaction between AMFP and TCNE. The measurements revealed that DCE was the most suitable solvent for both reactants and the AMFP–TCNE CT complex, as it provided excellent solvation as well as high and consistent absorbance.

### 2.2. Experimental Electronic Absorption Spectra (CT Band)

**Figure 1:**
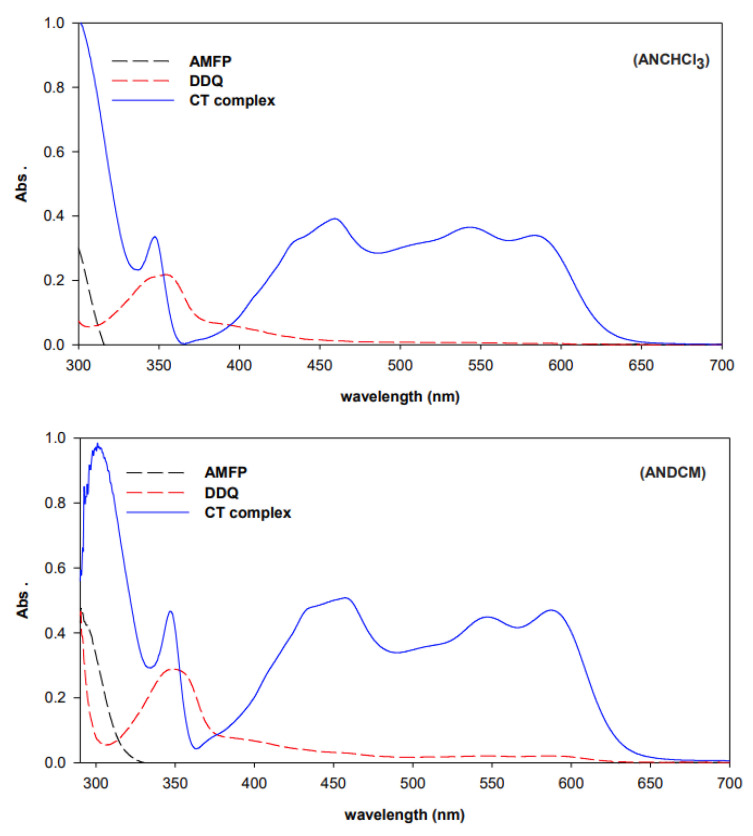
Electronic spectra of 1.0 × 10^−4^ mol L^−1^ AMFP, 1.0 × 10^−^^4^ mol L^−1^ DDQ, and (1:1) AMFP–DDQ CT complex in different solvents systems.

Figure 1 shows the electronic absorption spectra of 1.0 × 10^−4^ mol L^−1^ DDQ (pale yellow), 1.0 × 10^−4^ mol L^−1^ AMFP (colorless), and a (1:1) mixture of DDQ and AMFP (intense purple color) in two different solvents systems, ANCHCl_3_ and ANDCM, are measured in the region 300–700 nm. According to the spectra, when DDQ and AMFP are mixed, hyperchromic and bathochromic effects were observed, which could be attributed to CT complex formation.

**Scheme 1:**
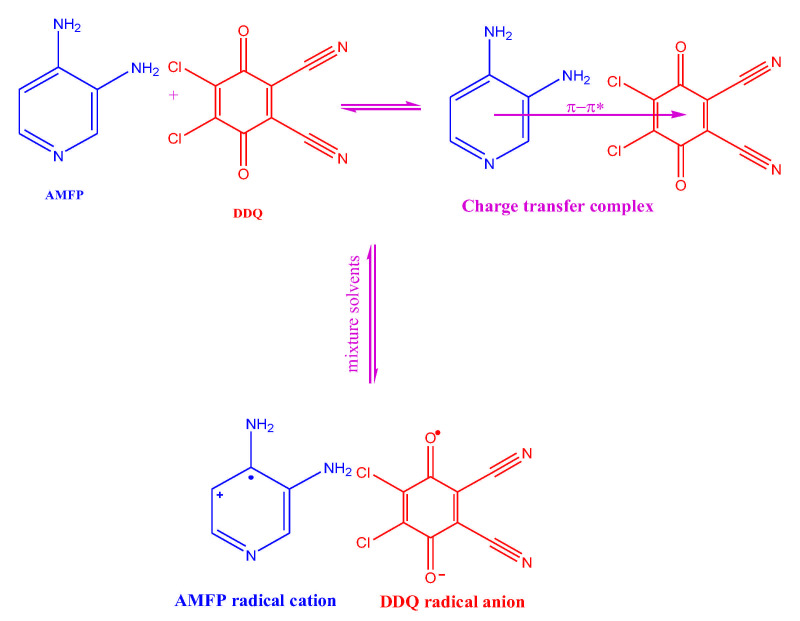
The suggested reaction of AMFP with DDQ in a polar/nonpolar mixture system.

It is related to π-π* electronic transitions, which have multi-CT bands at 584, 544, and 459 nm in the ANCHCl_3_ system and 587, 546, and 458.5 nm in the ANDCM system. The appearance of these multi-CT bands has been attributed to the electron transition from more than one closely located HOMO of AMFP to LUMO(s) of DDQ [35]. The *λ_max_* of one of the CT bands, i.e., 584 nm for the complex in ANCHCl_3_ and 587 nm in ANDCM, was chosen for quantitative measurements to provide the highest sensitivity. The radical anion of DDQ, which is intensely purple-colored, was produced in the studied solvents systems as a result of complete charge transfer from the donor (AMFP) to the acceptor (DDQ), as suggested in Scheme 1 [36,37]. This situation appears to be caused by the strong e-donating nature of AMFP, and the high electron affinity of DDQ (1.9 eV) [35].

**Figure 2:**
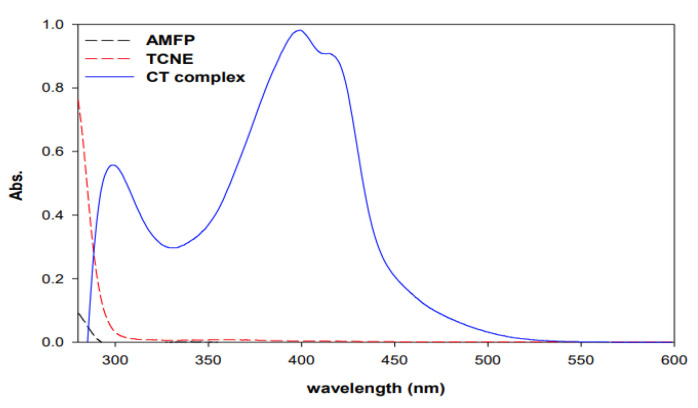
Electronic spectra of 1.0 × 10^−4^ mol L^−1^ AMFP, 1.0 × 10^−4^mol L^−1^ TCNE, and (1:1) AMFP–TCNE CT complex in DCE.

**Scheme 2:**
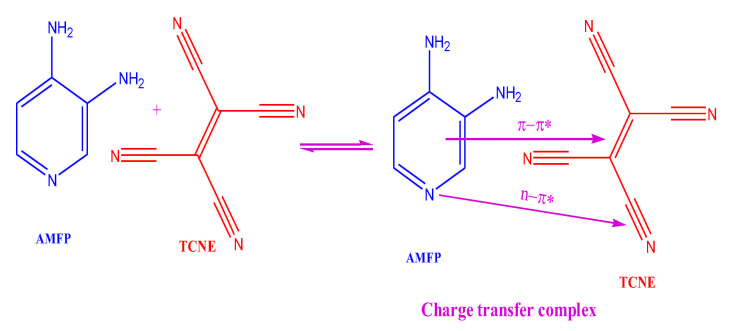
The suggested reaction of AMFP with TCNE in DCE solvent.

Figure 2 shows the electronic absorption spectra of the free reactants, AMFP and TCNE, and their mixture in DCE in the region 280–600 nm; 1.0 × 10^−4^ mol L^−1^ of TCNE solution in DCE has a pale-yellow color and an electronic spectrum that ranges from 300 to 450 nm. Aside from that, 1.0 × 10^−4^ mol L^−1^ of AMFP solution in DCE is colorless and shows no measurable absorption band in the same wavelength range. When TCNE was mixed with the AMFP solution, the color of the solution changed, indicating CT complex formation. The AMFP–TCNE complex’s electronic spectrum was characterized by high broadband in the visible region. This band is intense, high, and around 408 nm. The band head was split into two maxima (399 nm and 417 nm), indicating two interaction modes in the complex (π → π* and n → π*). The same behavior has been reported for different donors that reacted with TCNE in a 1:1 ratio [38] (Scheme 2). The second *λ_max_* value of the CT band, i.e., 417 nm, was chosen for further measurements to ensure the highest sensitivity.

**Figure 3:**
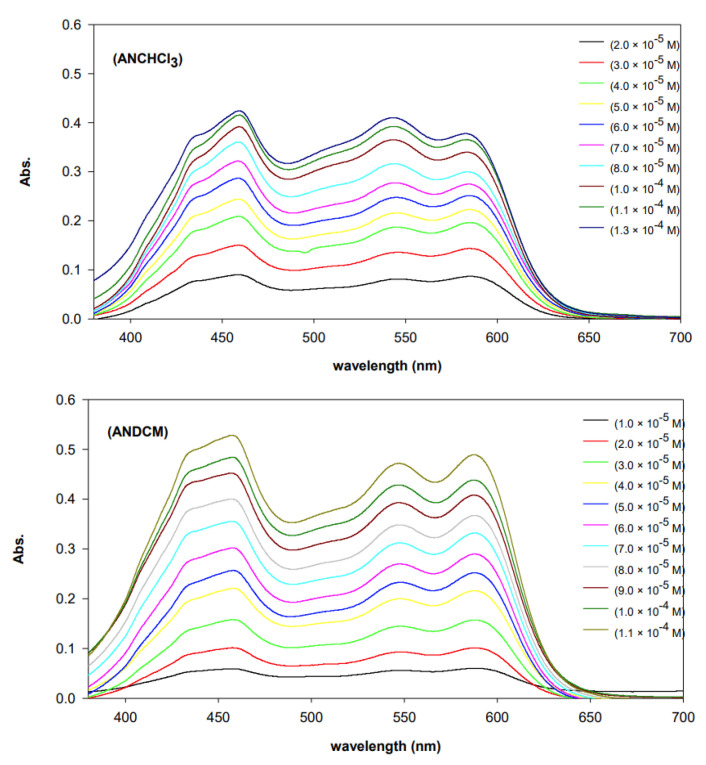
The electronic spectra of the AMFP–DDQ CT complex at different concentrations of AMFP in different solvents systems.

**Figure 4:**
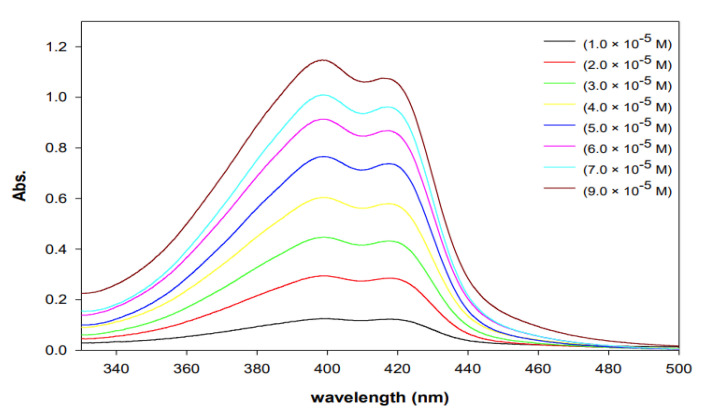
The electronic spectra of the AMFP–TCNE CT complex using different concentrations of AMFP in DCE.

Figure 3 and Figure 4 show the effect of increasing the concentration of AMFP on the absorbance of the AMFP–DDQ and AMFP–TCNE complexes, respectively. Figure 3 shows that the absorbance of the AMFP–DDQ complex increases with increasing AMFP concentration, indicating that the CT equilibrium is shifted toward the formation of radical anion and cation of DDQ and AMFP, respectively (Scheme 1) [37].

### 2.3. Molecular Composition of the CT Complexes

**Figure 5:**
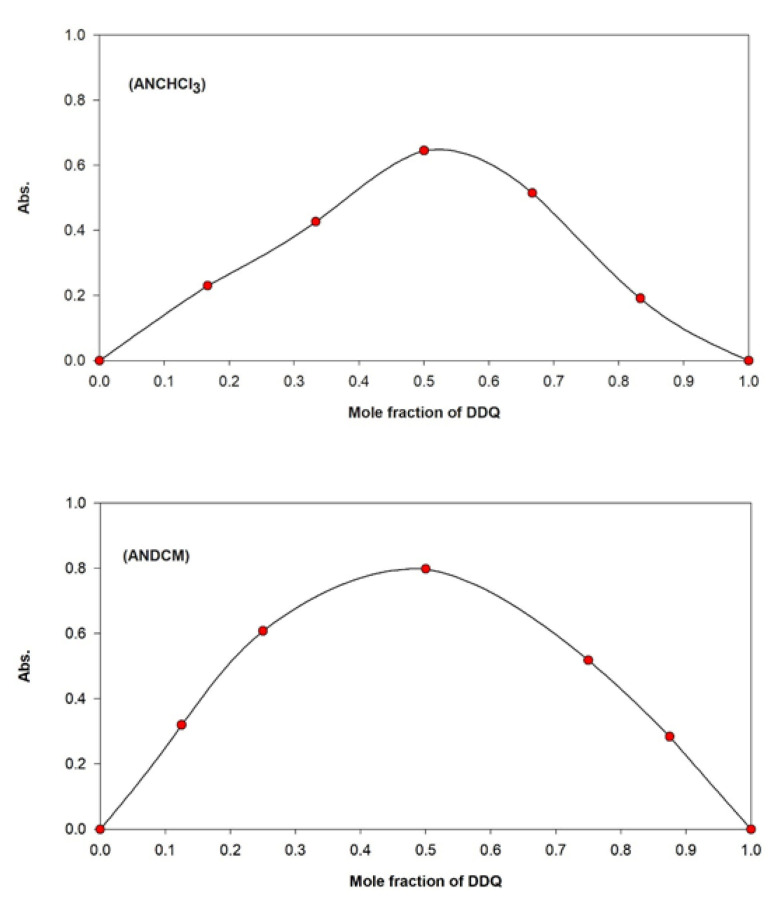
Job’s plots of AMFP–DDQ CT complex in different solvents systems.

**Figure 6:**
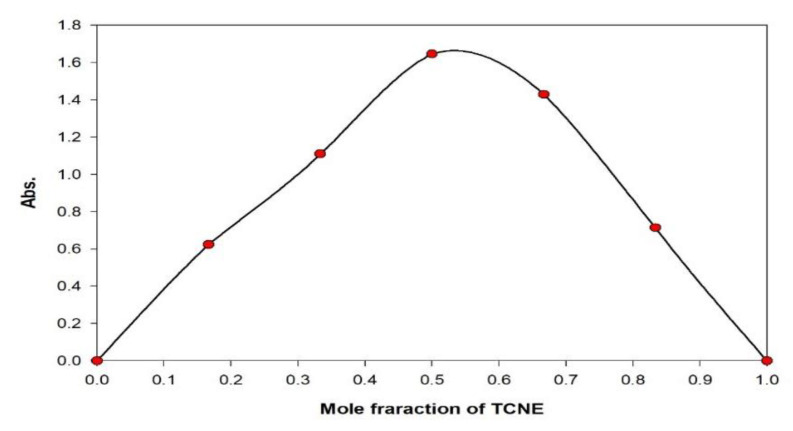
Job’s plot of AMFP–TCNE CT complex in DCE.

The AMFP–DDQ and AMFP–TCNE complexes’ molecular composition were estimated using Job’s method of continuous variations [39]. The relationship between the absorbance of the formed complex and the mole fraction of the acceptor was plotted (Figure 5 and Figure 6). From plots, the maximum absorbance of both complexes at *λ_max_* was observed at a 0.5-mole fraction of acceptor in the studied solvents, indicating a 1:1 CT complex in all cases.

### 2.4. The Effect of Temperature

**Figure 7:**
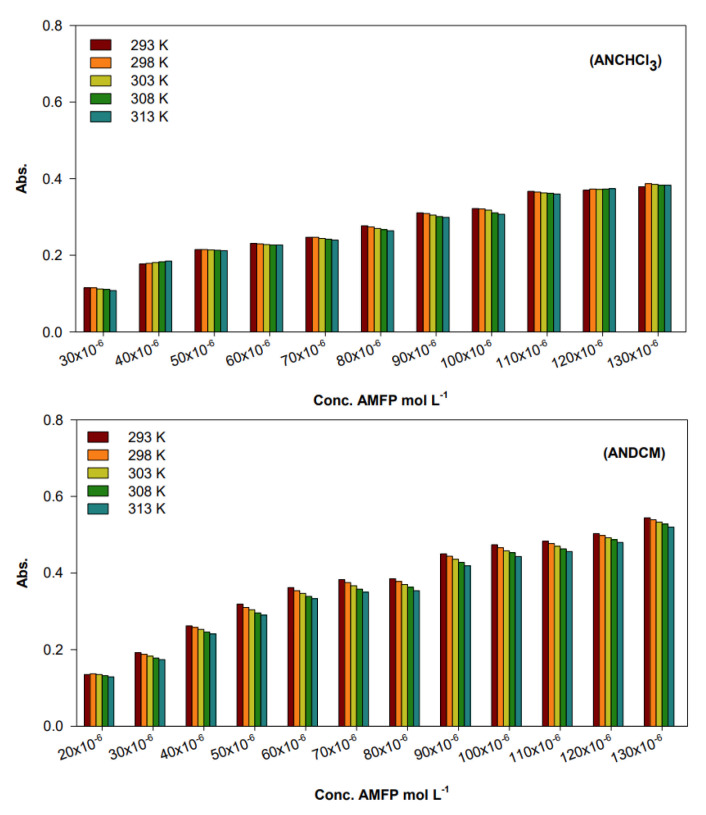
Effect of temperature on the absorbance of the AMFP–DDQ CT complex in different solvents systems.

The absorbance of the AMFP–DDQ complex was measured over a wide temperature range from 293 to 313 K by mixing a fixed concentration of DDQ (1.0 × 10^−4^ mol L^−1^) with different concentrations of AMFP. The optimum temperature was discovered to be 293 K, where the highest absorbance was recorded, as shown in Figure 7. Figure 7 shows that the absorbance of the AMFP–DDQ complex in ANCHCl_3_ was relatively constant at different temperatures.

**Figure 8:**
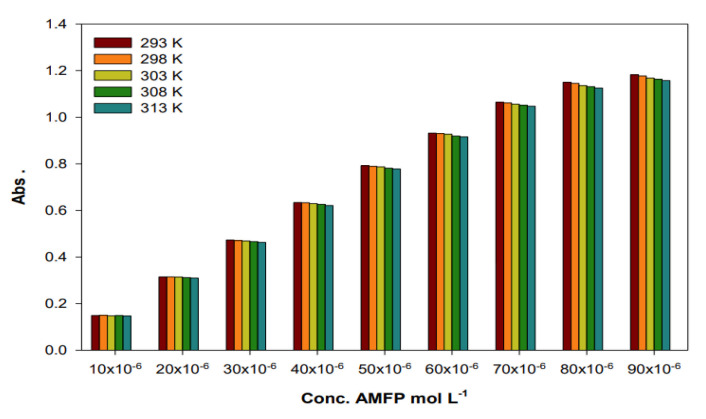
Effect of temperature on the absorbance of the AMFP–TCNE CT complex in DCE.

On the other hand, increasing temperatures decreased the absorbance of the AMFP–DDQ complex in ANDCM, which could be attributed to the dissociation of the reaction product from the reactants, as shown in Scheme 1. In addition, the absorbance of the AMFP–TCNE complex in DCE was measured at different temperatures (293–313 K), using a constant concentration of DDQ (1.0 × 10^−4^ mol L^−1^) with varying concentrations of AMFP (Figure 8), with 293 K as the optimum temperature. It was found that the absorbance of the formed complex was slightly reduced, specifically at higher temperatures (Figure 8).

### 2.5. Determination of Formation Constant (K_CT_) and Solvation Studies

Based on the spectral data listed in Table 1 and Table 2, the stability of the AMFP–DDQ and AMFP–TCNE CT complexes was investigated by estimating the formation constant, *K_CT_* (L mol^−1^), and the absorptivity coefficient, *ε_CT_* (L mol^−1^ cm^−1^), at different temperatures (293–313 K). The *K_CT_* and *ε_CT_* were calculated using the straight-line method from the Benesi–Hildebrand (HB) equation (Equation (1)) [40].

(1) $$\frac{\left[C_{A}\right]}{Abs}=\frac{1}{K_{CT}\cdot \varepsilon_{CT}}\cdot \frac{1}{\left[C_{D}\right]}+\frac{1}{\varepsilon_{CT}}$$

**Figure 9:**
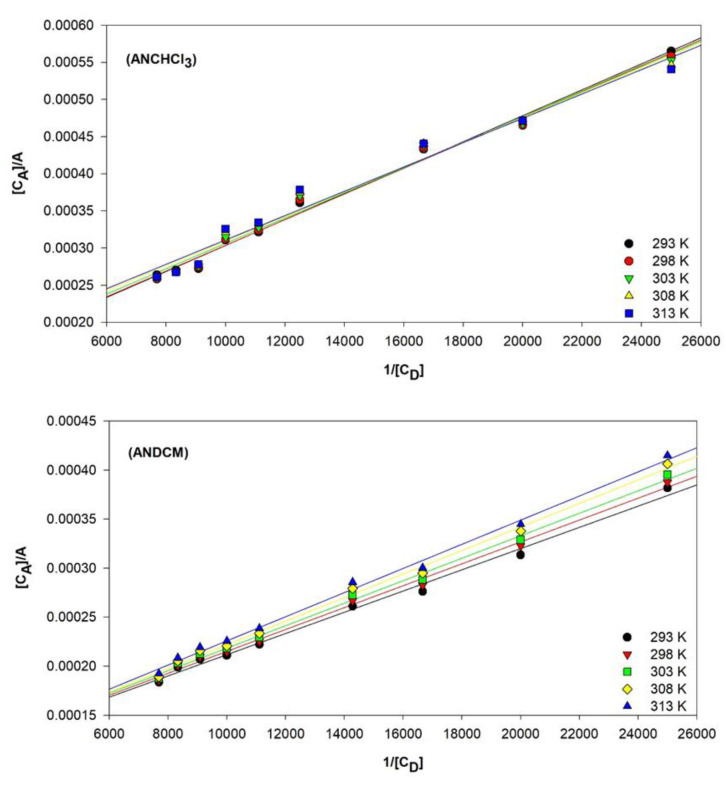
Benesi–Hildebrand plots of AMFP–DDQ CT complex in different solvent systems at different temperatures.

**Figure 10:**
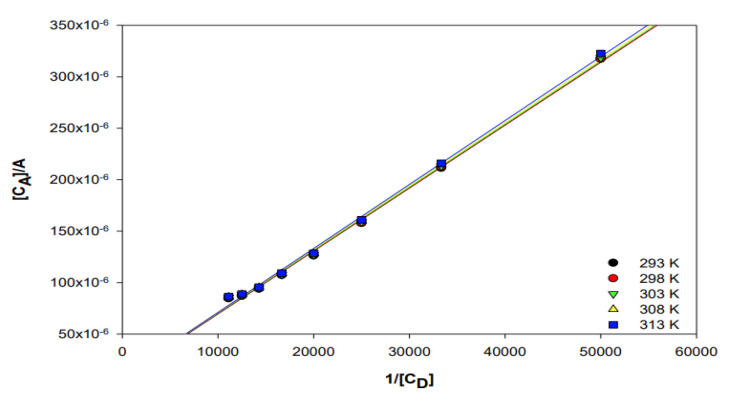
Benesi–Hildebrand plot of AMFP–TCNE CT complex in DCE at different temperatures.

Straight lines were obtained by plotting the values of [*C_A_*]/*Abs* against 1/[*C_D_*] for both CT complexes in different solvent systems (Figure 9 and Figure 10), where [*C_A_*] is the acceptor concentration (DDQ or TCNE), [*C_D_*] donor concentration (AMFP), and *Abs*. is the complex absorbance. The slopes and intercepts from the plots are equal to 1/*ε_CT_ K_CT_* and 1/*ε_CT_*, respectively, and the results are shown in Table 1 and Table 2.

As shown in Table 1 and Table 2, the values of *K_CT_* and *ε_CT_* for both complexes were high, confirming their stabilities. The *K_CT_* values of the AMFP–DDQ complex in both solvent systems are higher than the AMFP–TCNE complex in DCE, as shown in Table 1 and Table 2. These results suggest that *K_CT_* is highly dependent on the nature of the acceptor used. This phenomenon can be explained by the fact that DDQ is a non-aromatic compound which has two strong electron-withdrawing cyano groups, and when it is reduced by one electron, it acquires aromaticity. As a result, DDQ gains a large resonance energy, which explain its strong electron accepting properties. Hence, DDQ is a good e-acceptor in CT interactions. This phenomenon is related to the susceptibility of DDQ to one-electron reduction, i.e., reduction potential and LUMO energy level resulting in the high *K_CT_* for the AMFP–DDQ complex [41]. It is worth mentioning that the *K_CT_* for both complexes at different AMFP concentrations over the selected temperature range were constant in all studied solvents, indicating that the AMFP–DDQ complex is temperature-independent, as shown in Table 1 and Table 2.

Table 1 also shows how the *K_CT_* values of the AMFP–DDQ complex differ in both solvent systems. This variation in *K_CT_* values could be explained by the Kamlet–Taft solvent parameters *α* and *β* [42], as well as the electric permittivity of solvent (*ε_T_*), as shown in Table 3. As previously stated, AN is present in the same proportion in both solvent systems, so the difference in *K_CT_* values is frequently attributed to the other solvent in the system. As shown in Table 3, the lowest value of *K_CT_* was recorded in ANCHCl_3_, which can be explained by the *α* parameter of the solvents. The *α* value of CHCl_3_ in the first system (ANCHCl_3_) is 0.44, which is important in this situation because it leads to the solvation of the AMFP molecules. This solvation contains the C-H in CHCl_3_ and the lone pair of the nitrogen center in AMFP, resulting in a high steric hindrance and the lowest value of *K_CT_* in the ANCHCl_3_ system.

On the other hand, the *K_CT_* value of the AMFP–DDQ complex in ANDCM was nearly double that of ANCHCl_3_. Because no solvent–solute interactions take place in ANDCM, it appears that the low value of the *α* parameter of DCM (0.13) is responsible for this result [43]. The system’s polarity can also explain the difference in *K_CT_*. The ANDCM system is more polar (*ε_T_* for DCM = 8.93) than the ANCHCl_3_ system (*ε_T_* for CHCl_3_ = 4.90). The *K_CT_* values were increased by increasing the medium’s polarity, implying that the CT complex would be more stable in the more polar mixture than the less polar one. The low dielectric constant (*ε_T_*) of DCE (10.3) for the AMFP–TCNE complex leads to a stable interaction between the donor and acceptor’s molecular orbitals, resulting in a high value of *K_CT_*.

### 2.6. Calculation of Experimental Spectrophysical Parameters

The standard free energy change, Δ*G*° (kJ mol^−1^) of the CT interaction between AMFP and DDQ or TCNE was calculated using the following equation (Equation (2)) [44].

(2) $$∆G{}^{\circ}=-2.303RTlogK_{CT}$$

where *R* is the universal gas constant (8.314 mol^−1^ K), *T* is the absolute temperature (Kalvin), and *K_CT_* is the CT complex formation constant (L mol^−1^). Table 1 and Table 2 show the calculated values of Δ*G*° for AMFP CT complexes in different solvents systems at different temperatures. All Δ*G*° values are negative, confirming the spontaneous formation of [AMFP-DDQ] and [AMFP-TCNE] CT complexes in the solvent systems under investigation. Table 1 and Table 2 show that as the temperature rises, the Δ*G*° values become more negative as the components are subjected to more physical strain and less freedom [45].

The experimental oscillator strength (*f*), a dimensionless quantity, used to express the CT band’s transition probability [46] and the transition dipole moments (*μ_CT_*) [47] of the CT complexes were calculated at different temperatures using the following equations [48]:

(3) $$f=4.32\times 10^{-9}\left[\varepsilon_{max}\cdot ∆\nu_{\frac{1}{2}}\right]$$

(4) $$\mu_{CT}=0.0953{\left(\varepsilon_{max}\cdot \frac{∆\nu_{\frac{1}{2}}}{\nu_{max}}\right)}^{1/2}$$

where Δ*ν*_1/2_ is the half bandwidth of the absorbance in cm^−^^1^, and *ε_max_* and ν*_max_* are the molar extinction coefficient and wavenumber for the maximum absorption of the CT complexes, respectively. Table 4 shows the *f* and *μ_CT_* values of the AMFP–DDQ and AMFP–TCNE CT complexes in the studied solvents. The high values of *f* and *μ_CT_* indicate that AMFP has a strong CT interaction with both DDQ and TCNE in all studied solvents, with relatively high probabilities of CT transition between AMFP and the acceptors. It is worth noting that the AMFP–TCNE complex has higher *f* and *μ_CT_* values than the AMFP–DDQ complex. This is consistent with the *ε_CT_* values (Table 1 and Table 2), which express the probability of electron transition between the reactants, as it is higher for AMFP–TCNE (11 × 10^4^ L mol^−^^1^ cm^−^^1^) than AMFP–DDQ (10 × 10^3^ L mol^−^^1^ cm^−^^1^). The CT transition energy, *E_CT_* (eV), was calculated using Equation (5) [49], where *λ_max_* is the maximum wavelength of the formed CT complexes.

(5) $$E_{CT}=1243.667/\lambda_{max}$$

Table 4 shows the *E_CT_* values of both complexes in different solvents. The stability of the formed complex decreased as the *E_CT_* between donor and acceptor increased. As a result, the *E_CT_* of the AMFP–TCNE complex is higher than that of the AMFP–DDQ complex, which is consistent with the complex *K_CT_* values.

The electron-donating power can be evaluated by its ionization potential (*I_p_*) of the free donor (AMFP) in both CT complexes. *I_p_* is the energy required to ionize a molecule by removing an electron from it. The equation developed by Aloisi and Pignataro (Equation (6)) was used to calculate the values of *I_p_* [50].

(6) $$I_{P}\left(eV\right)=a+b\times 10^{-4}\cdot {\bar{\nu }}_{max}$$

where is ${\bar{\nu }}_{max}$ the wavenumber of the CT band in cm^−^^1^, *a* = 5.76 and *b* = 1.53 for DDQ, and *a* = 5.21 and *b* = 1.65 for TCNE. Table 4 shows the *I_p_* values of AMFP in AMFP–DDQ and AMFP–TCNE CT complexes in the studied solvent systems at different temperatures. The values were relatively low, indicating that AMFP has high donating power, thus high stability of the formed CT complexes. In addition, the *I_p_* values of the AMFP–DDQ complex in the studied systems are nearly identical and constant, confirming that an interaction between the same HOMO-donor-LUMO-acceptor forms the CT complex. In contacts, the *I_p_* for the AMFP–TCNE complex is higher than for the AMFP–DDQ complex, indicating that *I_p_* is affected by acceptor nature. It has been reported that the donor’s *I_p_* may be related to the complex’s CT transition energy [51] (Table 4).

The calculation of the dissociation energy, *W* (eV), which is the electrostatic energy of the ion pair [D^●+^, A^●−^]”, provides additional evidence of the nature of the CT interaction between AMFP and both acceptors in the studied systems. From the CT transition energy, the dissociation energy can be calculated as follows:

(7) $$E_{CT}=I_{P}-E_{A}-W$$

where *E_CT_* is the CT energy of the CT complexes, *I_p_* is the ionization potential of AMFP, and *E_A_* is the acceptor’s electron affinity. The *E_A_* for DDQ is 1.9 eV [35], and for TCNE is 3.17 eV [52]. Table 4 shows the values of *W* of both complexes, which are high and temperature independent. These results suggest that the AMFP–DDQ and AMFP–TCNE complexes are stable under the conditions investigated.

Finally, the resonance energy, *R_N_* (eV), of the CT complexes in the ground state is calculated using the Briegleb and Czekalla equation (Equation (8)) [53].

(8) $$R_{N}=\left[\varepsilon_{CT}\cdot h\nu_{CT}\right]/\left[7.7\times 10^{4}+3.5\varepsilon_{CT}\right]$$

Table 4 shows the calculated *R_N_* values, which are relatively high, indicating that the AMFP complex is strongly bound in the studied solvents and exhibits good resonance stabilization. These values were nearly identical at different temperatures, as shown in Table 4. It is worth noting that the *R_N_* mimicked the exact behavior of *f* and *μ_CT_* by recording a higher value for the AMFP–TCNE complex.

### 2.7. Quantitative Application of the CT Reaction

AMFP drug quantitative analysis in its pure form can be achieved by developing simple, rapid, and accurate spectrophotometric methods. These methods rely on CT complexation between AMFP and DDQ or TCNE in the solvent systems under consideration. Using various 1:1 molar ratios of AMFP to DDQ or TCNE, calibration curves were constructed. The regression equations of the calibration curves were calculated using the least-squares method [54]. Table 5 contains the statistical data for the regression equation. The calibration curves were linear over a wide range of AMFP concentrations; they were 1.0–7.6 and 0.5–7.0 μg mL^−1^ for [AMFP-DDQ] in ANCHCl_3_ and ANDCM, respectively, with good correlation coefficients. On the other hand, the linear range of the AMFP–TCNE calibration curve in DCE is 1.0–7.6 μg mL^−1^ and with good correlation coefficients (Table 5). The methods were validated by determining the LOD and LOQ values [54]. Table 5 shows that both LOD and LOQ recorded small values, confirming the high sensitivity of the suggested methods. Furthermore, it confirms that the AMFP–TCNE method is more sensitive for determining AMFP in its pure form. In addition, the values of the *S_a_*, *S_b_*, and *S_y/x_* were calculated and recorded in Table 5. The *S_a_*, *S_b_* values were low, confirming the excellent linearity between absorbance and concentration. Moreover, the low *S_y/x_* values indicate that the points are close to the straight lines in all systems.

The accuracy and precision of both methods were established by analyzing solutions containing five or six different AMFP concentrations (within the linear range). The measurements were repeated five times using the proposed methods for determining AMFP with DDQ or TCNE and measuring the absorbance of their CT complex in the different solvent systems. The concentration of AMFP was determined using the regression equations, and the recovery percentages (% Rec), relative standard deviation (% RSD), and relative error (% RE) were calculated. The results were compiled in Table 6, where the % Rec was close to 100%, and % RE values were low, indicating the high accuracy of the proposed methods. In addition, the % RSD values were low, confirming the high precision of the proposed methods for AMFP determination with DDQ or TCNE in all studied systems.

### 2.8. DFT Calculations

#### 2.8.1. Optimized Structures

**Figure 11:**
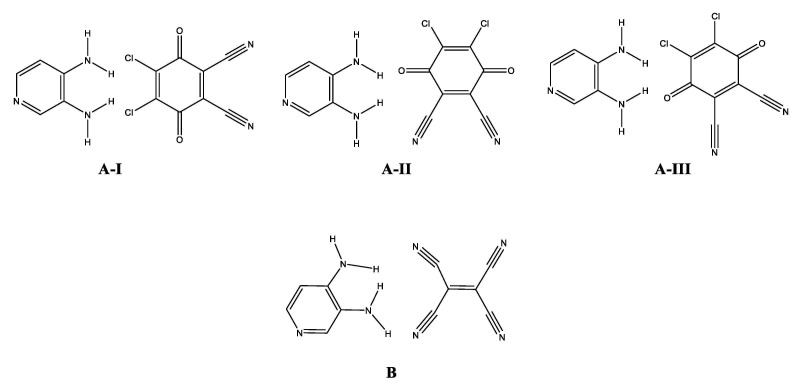
Proposed molecular structures of (**A**) AMFP–DDQ and (**B**) AMFP–TCNE CT complexes.

**Figure 12:**
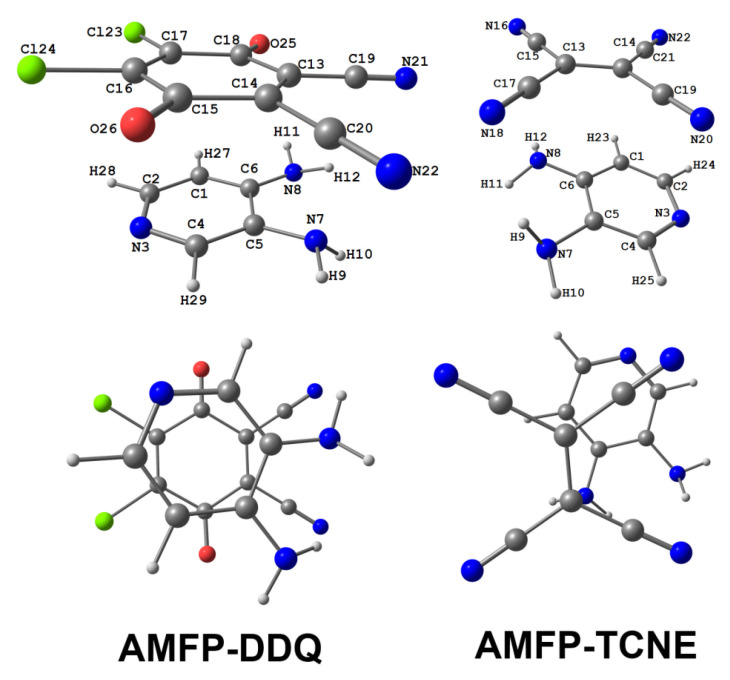
The atom numbering (**upper**) and the view showing the overlay between the donor and acceptor (**lower**) at the optimized molecular structures of the AMFP–DDQ and AMFP–TCNE CT complexes using the WB97XD method.

Figure 11 shows the suggested structures of the AMFP–DDQ and AMFP–TCNE CT complexes, where Figure 12 shows the optimized structures of the most stable structure. Some π-π* stacking interactions stabilize the CT complexes. There are four significant interactions for the AMFP–DDQ complex: C6…C14 (3.203 Å), C6…C13 (3.341 Å), C1…C16 (3.154 Å), and C1…C15 (3.268 Å). On the other hand, the C1…C14 (3.166 Å) and C6…C13 (3.225 Å) are the most important π-π* stacking interactions in the AMFP–TCNE complex. XYZ coordinates for the optimized geometries of the complexes can be found in the Supplementary materials. 

#### 2.8.2. Atomic Charge Distribution

The NBO method at the same level of theory was applied to calculate the charge populations at the different atomic sites, as the natural charges obtained from the NBO population analysis are less sensitive to basis set variations compared with Mulliken population analysis as example [55]. The AMFP–DDQ and AMFP–TCNE complexes comprise two fragments. The net natural charges are −0.1328 and −0.0706 e at the DDQ and TCNE fragments, respectively. The second fragment (AMFP) was predicted to have values of opposite signs. As a result, DDQ and TCNE are acceptors, while the AMFP is a donor. In this regard, it is possible to conclude that DDQ is a better acceptor than TCNE, which could be explained by the low lying π*-orbitals of the DDQ molecule.

#### 2.8.3. Determination of Reactivity Parameters

To understand various aspects of reactivity associated with chemical reactions, reactivity parameters such as ionization potential (*I_p_*), electron affinity (*A*), chemical potential (*μ*), hardness (*η*), and electrophilicity index (*ω*) were determined [56,57,58,59,60,61]. These parameters are defined in Equations (9)–(13), and their values for AMFP, DDQ, and TCNE are listed in Table 7.

*I_p_* = −*E_HOMO_* (9)

*A* = −*E_LUMO_* (10)

*η* = (*I_p_* − *A*)/2 (11)

*μ* = −*X* (12)

*ω* = *μ*^2^/2*η* (13)

The chemical species with the highest value of μ and HOMO energy is best suited as a donor. In contrast, the best acceptor has a low value of μ and a low LUMO energy level. In this regard, the AMFP is an e-donor fragment in both complexes, whereas the DDQ and TCNE species are acceptors (Table 7). The electrophilicity index (ω) is lower for the AMFP molecule and higher for DDQ and TCNE, supporting this conclusion.

#### 2.8.4. The Calculated Electronic Absorption Spectra (CT Band)

**Figure 13:**
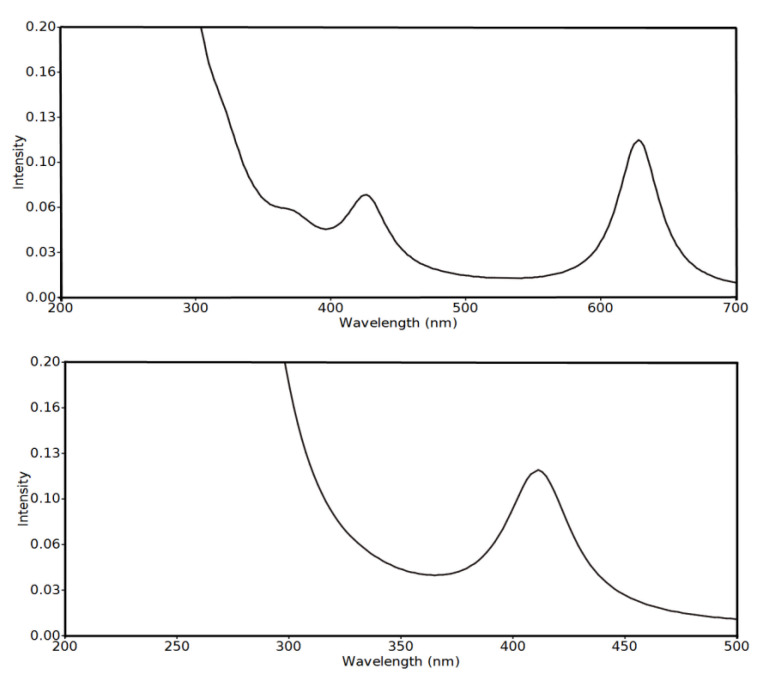
The calculated electronic spectra of the AMFP–DDQ (upper) and AMFP–TCNE (lower) CT complexes.

**Figure 14:**
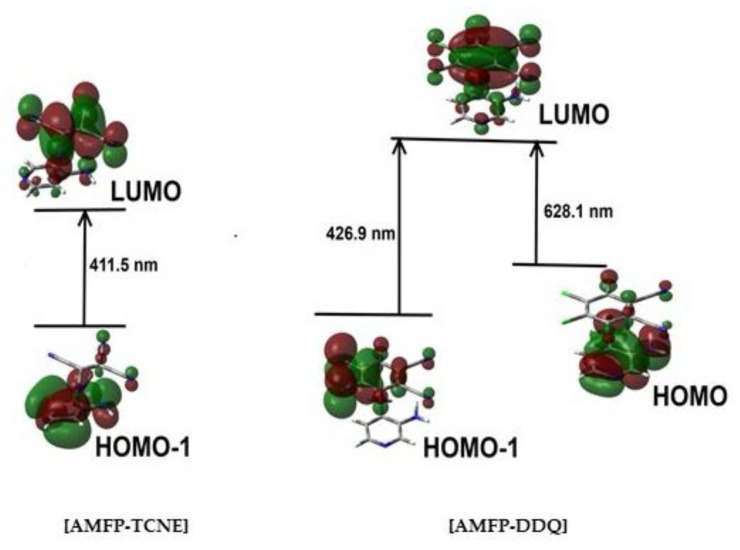
The molecular orbitals contributed to the electronic absorption of the AMFP–DDQ and AMFP–TCNE CT complexes.

TD-DFT calculations were used to compute the electronic spectra of the AMFP–DDQ and AMFP–TCNE CT complexes in CHCl_3_ as a solvent (Figure 13). The AMFP–DDQ complex was predicted to have two visible bands at 426.9 nm (exp. 459 nm) and 628.1 nm (exp. 584 nm) by TD-DFT calculations. Their oscillator strengths were calculated as 0.054 and 0.111, respectively, and theoretically assigned to HOMO-1 → LUMO and HOMO → LUMO excitations. On the other hand, the AMFP–TCNE complex showed a double split band in the experimental spectra observed at 399 and 417 nm. The TD-DFT calculations for this complex predicted the longest wavelength band at 411.5 nm with oscillator strengths (f) of 0.105 assigned to HOMO-1 → LUMO excitation (Figure 14). The electronic transition from the AMFP molecule’s HOMO as a donor to the TCNE fragment’s LUMO as an acceptor, indicating a CT-based transition. The former could primarily be assigned to an internal electronic transition within the DDQ fragment (Figure 14). In contrast, the longer wavelength band could be assigned to the electronic transition from the AMFP molecule’s HOMO as a donor to the DDQ fragment’s LUMO as an acceptor, indicating a CT-based transition [34].

#### 2.8.5. Natural Bond Orbital (NBO) Analysis

The NBO calculations provide a useful quantitative expression for the strength of electron delocalization processes between electron-pair occupied NBOs and the empty antibonding NBOs. Table 8 shows the calculated results of the stabilization energies (E^(2)^) of the different electron delocalization processes in the studied systems. In the AMFP–DDQ system, the electron donor fragment (AMFP) stabilized the system by many π → π*, n → π* and n → σ* intramolecular CT processes up to 44.24 kcal mol^−1^ (BD(2)C5-C6 → BD*(2)N3-C4), 32.46 kcal mol^−1^ (LP(1)N8 → BD*(2)C5–C6), and 12.09 kcal mol^−1^ (LP(1)N3 → BD*(1)C4–C5), respectively. The corresponding values in the AMFP–TCNE system are 45.49 kcal mol^−1^ (BD(2) C1–C6 → BD*(2)C2–N3), 36.15 kcal mol^−1^ (LP(1) N8 → BD*(2) C1–C6), and 11.72 kcal mol^−1^ (LP(1) N3 → BD*(1) C1–C2), respectively.

On the other hand, the number of intramolecular CT processes that occurred in the electron acceptor fragment of the AMFP–DDQ complex is larger than that of the AMFP–TCNE system, due to the presence of a more extended π-system in the AMFP–DDQ than in the AMFP–TCNE. The maximum stabilization energies (E^(2)^) in the AMFP–DDQ complex are 20.87 kcal mol^−1^ (BD(2) C16-C17 → BD*(2) C18-O25), 29.34 kcal mol^−1^ (LP(2)O26 → BD*(1)C15-C16), and 27.41 kcal mol^−1^ (LP(3)Cl24 → BD*(2)C16-C17). In the AMFP–TCNE, the BD(2)C13-C14 → BD*(3)C17-N18 (17.88 kcal mol^−1^), LP(1)N20 → BD*(1)C14-C19 (15.19 kcal mol^−1^), and BD(1)C19-N20 → BD*(1)C14-C19 (6.47 kcal mol^−1^) are the strongest π → π*, n → σ* and σ → σ* intramolecular CT processes that occurred in the TCNE fragments.

## 3. Experimental

### 3.1. Materials and Stock Solutions

The donor (AMFP) and acceptors (DDQ and TCNE) were purchased from Sigma-Aldrich USA (98%). All organic solvents (99.9%) were obtained from Fisher (Honeywell) and used as received. Standard stock solutions of AMFP (1.0 × 10^−3^ mol L^−1^), DDQ (1.0 × 10^−3^ mol L^−1^), and TCNE (1.0 × 10^−3^ mol L^−1^) were prepared by dissolving the appropriate weight of each in separate volumetric flasks of 50 mL using the selected solvent. Stock solutions of donor and acceptors were used for further measurements by diluting with solvent.

### 3.2. Spectroscopy Measurements

The electronic absorption spectra of free AMFP, free acceptors (DDQ and TCNE), and the corresponding CT complexes were recorded in the 280–700 nm regions using a Shimadzu 1800 UV–Vis spectrophotometer equipped with 1.0 cm quartz cells at room temperature. The blank used for the free reactants was the solvent, where the acceptor solution was used as blank for the corresponding CT complex spectrum to eliminate any overlap between the complex and the acceptor bands.

For spectral determination of the formation constant (*K_CT_*), the Benesi–Hildebrand method [40] has been applied according to the following procedure: 1 mL of 1.0 × 10^−3^ mol L^−1^ stock solution of the acceptor (DDQ or TCNE) was transferred to a series of 10 mL volumetric flasks. To each of these flasks, different concentrations of AMFP were added from the stock solution (1.0 × 10^−3^ mol L^−1^). The volume was made up to the mark with solvent. The spectra of all solutions were recorded at different temperatures (293, 298, …, 313 K) using a Shimadzu TCC-ZUOA temperature controller unit.

### 3.3. Determination of Molecular Composition

The molecular composition of the CT interaction between AMFP and both DDQ and TCNE was determined by applying Job’s method of continuous variations [39]. In this method, different volumes of 1.0 × 10^−3^ mol L^−1^ AMFP and DDQ or TCNE were mixed, but the sum volume was kept constant in 5 mL volumetric flasks. The electronic absorbance of all AMFP-acceptor systems was measured at *λ_max_*. The absorbance values were plotted against the molar fraction of the acceptor.

### 3.4. Computational Details

The WB97XD/6-31++G(d,p) method, with the aid of Gaussian 09 software, was used to compute the optimized structures of AMFP–DDQ (A) and AMFP–TCNE (B) complexes, and all optimized structures gave no imaginary vibrational modes [62]. GaussView 4.1 and Chemcraft programs [63,64] were used to extract the computational results. The natural bond orbital (NBO) method was applied to compute the charge distribution on the atomic sites [65,66]. The TD-DFT method was used to deduce the origin electronic spectra, while accounting for solvent effects (CHCl_3_) using the polarizable continuum model (PCM) [67].

## 4. Conclusions

AMFP is a drug that is used to treat LEMS disease. CT complexation formed between AMFP and two π-acceptors, DDQ and TCNE in different solvents, and a mixture of solvents systems were studied experimentally and theoretically. The formation of the CT complexes was confirmed by the appearance of new absorption bands in the visible region. Using the continuous variations method, the molecular composition of both CT complexes is determined to be 1:1. The investigated complexes’ formation constants (*K_CT_*) and molar extinction coefficient (*ε_CT_*) were calculated at different temperatures using the Bensi–Hildebrand straight-line method. The high *K_CT_* values confirmed the high stability of the studied complexes. *K_CT_* for both complexes was discovered to be temperature independent. The effect of solvent systems on the stability of the AMFP–DDQ complex was investigated and discussed. Simple, rapid, and accurate spectrophotometric methods for determining AMFP in pure form have been proposed and statically validated using the CT reaction between AMFP and DDQ or TCNE in various solvent systems. The obtained results show that the applied methods for determining AMFP in its pure form, particularly the AMFP–TCNE method, has a high degree of accuracy and precision.

Using DFT calculations, the DDQ and TCNE fragments serve as electron acceptors, while AMFP serves as an electron donor. The TD-DFT calculations predicted two visible bands at 426.9 nm and 628.1 nm for the AMFP–DDQ complex, assigned to HOMO-1 → LUMO and HOMO → LUMO excitations, respectively, which could primarily be assigned as an internal electronic transition within the DDQ fragment and a charge transfer-based transition. Moreover, a charge transfer-based transition (HOMO-1 → LUMO) was predicted at 411.5 nm for the AMFP–TCNE complex. The stabilization energies in CT complexes were also compared to those in free molecules and discussed based on NBO calculations.

## Figures and Tables

**Table 1:** Benesi–Hildebrand spectral data of AMFP–DDQ CT complex in different solvent systems.

ANCHCl_3._		Abs. at l_max_ = 584 nm				
[C_A_] mol L^−1^	[C_D_] mol L^−1^	293K	298K	303K	308K	313K
1.0 × 10^−4^	2.0 × 10^−5^	0.082	0.084	0.092	0.095	0.098
1.0 × 10^−4^	3.0 × 10^−5^	0.115	0.115	0.112	0.111	0.108
1.0 × 10^−4^	4.0 × 10^−5^	0.177	0.179	0.181	0.183	0.185
1.0 × 10^−4^	5.0 × 10^−5^	0.215	0.215	0.214	0.213	0.212
1.0 × 10^−4^	6.0 × 10^−5^	0.231	0.23	0.228	0.227	0.227
1.0 × 10^−4^	7.0 × 10^−5^	0.247	0.247	0.244	0.242	0.24
1.0 × 10^−4^	8.0 × 10^−5^	0.277	0.274	0.27	0.267	0.264
1.0 × 10^−4^	9.0 × 10^−5^	0.311	0.309	0.305	0.301	0.299
1.0 × 10^−4^	1.0 × 10^−4^	0.322	0.321	0.318	0.311	0.307
1.0 × 10^−4^	1.1 × 10^−4^	0.367	0.365	0.363	0.362	0.36
1.0 × 10^−4^	1.2 × 10^−4^	0.37	0.373	0.372	0.373	0.374
1.0 × 10^−4^	1.3 × 10^−4^	0.379	0.387	0.385	0.383	0.383
Formation constant (*K_CT_*)		5.0 × 10^3^	5.0 × 10^3^	5.0 × 10^3^	5.0 × 10^3^	5.0 × 10^3^
Absorptivity coefficient (*ε_CT_*)		10 × 10^3^	10 × 10^3^	10 × 10^3^	10 × 10^3^	10 × 10^3^
Correlation coefficient (*R*^2^)		0.991	0.988	0.983	0.976	0.97
−Δ*G*° (kJ mol^−1^)		20.751	21.105	21.459	21.814	22.168
**ANDCM**		**Abs. at *λ_max_* = 587 nm**				
[C_A_] mol L^−^^1^	[C_D_] mol L^−^^1^	293K	298K	303K	308K	313K
1.0 × 10^−4^	2.0 × 10^−5^	0.135	0.137	0.135	0.132	0.129
1.0 × 10^−4^	3.0 × 10^−5^	0.192	0.188	0.183	0.178	0.174
1.0 × 10^−4^	4.0 × 10^−5^	0.262	0.258	0.253	0.246	0.241
1.0 × 10^−4^	5.0 × 10^−5^	0.319	0.310	0.304	0.296	0.290
1.0 × 10^−4^	6.0 × 10^−5^	0.362	0.354	0.347	0.339	0.333
1.0 × 10^−4^	7.0 × 10^−5^	0.383	0.375	0.367	0.358	0.350
1.0 × 10^−4^	8.0 × 10^−5^	0.385	0.378	0.370	0.363	0.354
1.0 × 10^−4^	9.0 × 10^−5^	0.450	0.444	0.436	0.428	0.419
1.0 × 10^−4^	1.0 × 10^−4^	0.474	0.466	0.458	0.453	0.443
1.0 × 10^−4^	1.1 × 10^−4^	0.483	0.477	0.470	0.463	0.456
1.0 × 10^−4^	1.2 × 10^−4^	0.503	0.498	0.492	0.487	0.480
1.0 × 10^−4^	1.3 × 10^−4^	0.544	0.539	0.533	0.528	0.520
Formation constant (*K_CT_*)		10 × 10^3^	10 × 10^3^	10 × 10^3^	10 × 10^3^	10 × 10^3^
Absorptivity coefficient (*ε_CT_*)		10 × 10^3^	10 × 10^3^	10 × 10^3^	10 × 10^3^	10 × 10^3^
Correlation coefficient (*R*^2^)		0.993	0.995	0.996	0.996	0.995
−Δ*G*° (kJ mol^−1^)		22.441	22.823	23.206	23.589	23.972

**Table 2:** Benesi–Hildebrand spectral data of AMFP–TCNE CT complex in DCE.

[C_A_] mol L^−1^	[C_D_] mol L^−1^	Abs. at *λ_max_* = 417 nm				
		293 K	298 K	303 K	308 K	313 K
1.0 × 10^−4^	1.0 × 10^−5^	0.149	0.15	0.148	0.149	0.147
1.0 × 10^−4^	2.0 × 10^−5^	0.315	0.315	0.314	0.312	0.31
1.0 × 10^−4^	3.0 × 10^−5^	0.471	0.471	0.469	0.466	0.463
1.0 × 10^−4^	4.0 × 10^−5^	0.634	0.633	0.629	0.626	0.621
1.0 × 10^−4^	5.0 × 10^−5^	0.789	0.789	0.787	0.781	0.778
1.0 × 10^−4^	6.0 × 10^−5^	0.932	0.93	0.927	0.919	0.916
1.0 × 10^−4^	7.0 × 10^−5^	1.064	1.061	1.056	1.051	1.047
1.0 × 10^−4^	8.0 × 10^−5^	1.147	1.145	1.136	1.131	1.125
1.0 × 10^−4^	9.0 × 10^−5^	1.179	1.177	1.168	1.163	1.157
Formation constant (*K_CT_*)		1.5 × 10^3^	1.5 × 10^3^	1.5 × 10^3^	1.5 × 10^3^	1.5 × 10^3^
Absorptivity coefficient (*ε_CT_*)		11 × 10^4^	11 × 10^4^	11 × 10^4^	11 × 10^4^	11 × 10^4^
Correlation coefficient (*R*^2^)		0.997	0.997	0.997	0.997	0.997
−Δ*G*° (kJ mol^−1^)		17.818	18.122	18.426	18.73	19.034

**Table 3:** Solvent parameters of the formation constant and the molar extinction coefficient of the AMFP–DDQ CT complex in different solvents systems.

Solvents	*K_CT_* at 293 K	*ε_CT_* at 293 K	*ε* *_Τ_*	*α*	*β*
ANCHCl_3_	5.0 × 10^3^	10 × 10^3^	-	-	-
ANDCM	10 × 10^3^	10 × 10^3^	-	-	-
CHCl3	-	-	4.81	0.44	0.1
DCM	-	-	8.93	0.13	0.1
AN	-	-	37.5	0.19	0.31

**Table 4:** The spectroscopic data of the CT complexes in different solvents systems at different temperatures.

Acceptor	Solvent Systems	Temp	*I_P_* (eV)	*f*	*µ_CT_* (Debye)	*E_CT_* (eV)	*W* (eV)	*R_N_* (eV)
DDQ	ANCHCl_3_	293 k	8.38	5.038	25	2.13	4.35	0.071
		298 k	8.38	5.144	25.26		4.35	0.073
		303 k	8.38	5.118	25.2		4.35	0.072
		313 k	8.38	5.091	25.13		4.35	0.072
	ANDCM	293 k	8.37	7.231	30.03	2.119	4.35	0.097
		298 k	8.37	7.165	29.98		4.35	0.096
		303 k	8.37	7.085	29.72		4.35	0.095
		313 k	8.37	7.018	29.58		4.35	0.094
TCNE	DCE	293 K	9.17	35.46	56.05	2.98	3.28	0.32
		298 K	9.17	35.31	55.93		3.28	0.32
		303 K	9.17	35.04	55.72		3.28	0.32
		308 K	9.17	34.89	55.6		3.28	0.32
		313 K	9.17	34.71	55.45		3.28	0.31

**Table 5:** Statistical data for the regression equation of AMFP determination methods.

Parameters	AMFP–DDQ Method		AMFP–TCNE Method
	ANCHCl_3_	ANDCM	DCE
Beer’s law limits, µg mL−1	1.0–7.6	0.5–7.0	0.1–7.6
LOD, µg mL^−1^	0.5085	0.4082	0.2726
LOQ, µg mL^−1^	1.5411	1.237	0.8261
Regression equation	Y = 0.0491x − 0.0553	Y = 0.0595x + 0.0267	Y = 0.1237x + 0.0152
Intercept, a ± *S_a_*	−0.0553 ± 0.0044	0.0267 ± 0.0074	0.0152 ± 0.0102
Slope, b ± *S_b_*	0.0491 ± 0.0009	0.0595 ± 0.0015	0.1237 ± 0.0023
*S_y/x_*	0.0053	0.0135	0.0206
Correlation coefficient, R	0.999	0.996	0.9978

**Table 6:** Accuracy and precision for the AMFP determination methods.

Acceptor	Solvent System	Amount Taken, µg mL^−1^	Amount Found, µg mL^−1^	^a^ % Rec	^b^ % RSD	^c^ % RE	^d^ Confidence Limits
DDQ	ANCHCl_3_	2.18	2.17	99.47	1.95	0.52	99.47 ± 0.05
		3.27	3.23	98.69	0.82	1.31	98.69 ± 0.03
		4.37	4.36	99.8	0.58	0.2	99.80 ± 0.03
		5.46	5.38	98.66	1.68	1.34	98.66 ± 0.11
		6.55	6.49	99.15	0.22	0.85	99.15 ± 0.02
	ANDCM	2.73	2.71	99.26	0.32	0.74	99.26 ± 0.01
		3.82	3.86	101.1	1.1	1.07	101.1 ± 0.05
		4.37	4.35	99.68	1.16	0.32	99.68 ± 0.06
		4.91	4.97	101.2	0.36	1.24	101.2 ± 0.02
		5.46	5.64	100.1	1.54	0.05	100.1 ± 0.10
		7.09	7.12	100.3	1.9	0.3	100.3 ± 0.17
TCNE	DCE	3.27	3.22	98.24	1.03	1.76	98.24 ± 0.04
		3.82	3.8	99.5	0.66	0.5	99.50 ± 0.03
		4.91	5.05	102.8	1.33	2.8	102.8 ± 0.08
		6.00	5.99	99.72	1.81	0.28	99.72 ± 0.13
		6.55	6.45	98.51	0.53	1.49	98.51 ± 0.04

^a^ % Rec = Recovery percentage, ^b^ % RSD = relative standard deviation, ^c^ % RE = relative error. ^d^ $\bar{X}=\frac{S\;t_{n-1\;}}{\sqrt{n}}$, *t_n_*_−1_ = 2.75 for *n* = 5 at 95% confidence level.

**Table 7:** Reactivity descriptors (eV) of the free DDQ, TCNE, and AMFP using the WB97XD method.

Parameter	DDQ	TCNE	AMFP
HOMO	−10.604	−11.410	−7.345
LUMO	−3.594	−3.432	0.782
*I_p_*	10.604	11.410	7.345
*A*	3.594	3.432	−0.782
*μ*	−7.099	−7.421	−3.282
*η*	3.505	3.989	4.063
*ω*	7.190	6.902	1.325

**Table 8:** Stabilization energies of donor-acceptor interactions in the AMFP–DDQ and AMFP–TCNE complexes.

Donor Orbital	Acceptor Orbital	E^(2)^	Donor Orbital	Acceptor Orbital	E^(2)^
AMFP–DDQ			AMFP–TCNE		
AMFP-Fragment					
BD(2) C1-C2	BD*(2) N3-C4	28.56	BD(2) C1-C6	BD*(2) C2-N3	45.49
BD(2) C1-C2	BD*(2) C5-C6	35.64	BD(2) C1-C6	BD*(2) C4-C5	24.90
BD(2) N3-C4	BD*(2) C1-C2	39.64	BD(2) C2-N3	BD*(2) C1-C6	21.36
BD(2) N3-C4	BD*(2) C5-C6	21.68	BD(2) C2-N3	BD*(2) C4-C5	40.47
BD(2) C5-C6	BD*(2) C1-C2	28.38	BD(2) C4-C5	BD*(2) C1-C6	38.36
BD(2) C5-C6	BD*(2) N3-C4	44.24	BD(2) C4-C5	BD*(2) C2-N3	28.12
LP(1) N3	BD*(1) C1-C2	11.56	LP(1) N3	BD*(1) C1-C2	11.72
LP(1) N3	BD*(1) C4-C5	12.09	LP(1) N3	BD*(1) C4-C5	11.45
LP(1) N7	BD*(2) C5-C6	29.16	LP(1) N7	BD*(2) C4-C5	17.85
LP(1) N8	BD*(2) C5-C6	32.46	LP(1) N8	BD*(2) C1-C6	36.15
DDQ–Fragment			TCNE-Fragment		
BD(1) C13-C19	BD*(1) C19-N21	5.870	BD(2) C13-C14	BD*(3) C15-N16	17.53
BD(1) C14-C20	BD*(1) C20-N22	5.990	BD(2) C13-C14	BD*(3) C17-N18	17.88
BD(1) C15-C16	BD*(1) C17-Cl23	6.190	BD(2) C13-C14	BD*(3) C19-N20	16.72
BD(1) C17-C18	BD*(1) C16-Cl24	6.030	BD(2) C13-C14	BD*(3) C21-N22	17.05
BD(1) C20-N22	BD*(1) C14-C20	6.290	BD(3) C15-N16	BD*(2) C13-C14	13.34
BD(2) C13-C14	BD*(2) C15-O26	18.64	BD(3) C17-N18	BD*(2) C13-C14	12.51
BD(2) C13-C14	BD*(2) C18-O25	18.97	BD(3) C19-N20	BD*(2) C13-C14	14.57
BD(2) C13-C14	BD*(3) C19-N21	17.90	BD(3) C21-N22	BD*(2) C13-C14	13.70
BD(2) C13-C14	BD*(3) C20-N22	17.20	BD(1) C13-C14	BD*(1) C17-N18	5.060
BD(2) C15-O26	BD*(2) C13-C14	7.780	LP(1) N16	BD*(1) C13-C15	14.95
BD(2) C13-C14	BD*(2) C16-C17	7.170	LP(1) N18	BD*(1) C13-C17	15.02
BD(2) C16-C17	BD*(2) C15-O26	19.45	LP(1) N20	BD*(1) C14-C19	15.19
BD(2) C16-C17	BD*(2) C18-O25	20.87	LP(1) N22	BD*(1) C14-C21	14.98
BD(2) C18-O25	BD*(2) C13-C14	7.120	BD(1) C13-C17	BD*(1) C17-N18	5.960
BD(2) C18-O25	BD*(2) C16-C17	6.750	BD(1) C14-C19	BD*(1) C19-N20	5.900
BD(2) C13-C14	BD*(2) C15-O26	18.64	BD(1) C14-C21	BD*(1) C21-N22	5.730
BD(3) C19-N21	BD*(2) C13-C14	13.44	BD(1) C15-N16	BD*(1) C13-C15	6.240
BD(3) C20-N22	BD*(2) C13-C14	13.77	BD(1) C17-N18	BD*(1) C13-C17	6.250
LP(1) N21	BD*(1) C13-C19	14.40	BD(1) C19-N20	BD*(1) C14-C19	6.470
LP(1) N22	BD*(1) C14-C20	14.46	BD(1) C21-N22	BD*(1) C14-C21	6.130
LP(2)Cl23	BD*(1) C16-C17	5.610			
LP(2)Cl23	BD*(1) C17-C18	6.980			
LP(3)Cl23	BD*(2) C16-C17	26.20			
LP(2)Cl24	BD*(1) C15-C16	7.310			
LP(2)Cl24	BD*(1) C16-C17	5.720			
LP(3)Cl24	BD*(2) C16-C17	27.41			
LP(2) O25	BD*(1) C13-C18	29.16			
LP(2) O25	BD*(1) C17-C18	28.92			
LP(2) O26	BD*(1) C14-C15	29.29			
LP(2) O26	BD*(1) C15-C16	29.34			
LP(2) O26	BD*(3) C19-N21	12.00			
LP(2) O26	BD*(3) C20-N22	10.93			

BD* is antibonding orbital.

## Data Availability

Not applicable.

## Supplementary Materials

The XYZ coordinates of the optimized geometry.

## Sample Availability

Samples of the compounds are commercially available.
