# Supplementary material for: New Charge Transfer Complexes of K+-Channel-Blocker Drug (Amifampridine; AMFP) for Sensitive Detection; Solution Investigations and DFT Studies
Source: Molecules. 2021 Oct 5;26(19):6037. doi: 10.3390/molecules26196037 (PMC8512129; doi:10.3390/molecules26196037)
Supplement: Supplementary file 1 [file molecules-26-06037-s001.zip › molecules-1383356-supplementary.pdf]

## The XYZ coordinates of the optimized geometry for AMFP-DDQ in gas phase

| | | | |
|---|---|---|---|
| C | -0.242004000 | -2.381635000 | 1.174082000 |
| C | -1.111919000 | -1.643656000 | 1.970921000 |
| N | -0.769007000 | -0.515273000 | 2.591957000 |
| C | 0.501112000 | -0.114203000 | 2.456196000 |
| C | 1.463053000 | -0.777746000 | 1.701513000 |
| C | 1.073268000 | -1.941725000 | 1.004352000 |
| N | 2.758682000 | -0.271787000 | 1.501127000 |
| N | 1.991645000 | -2.610576000 | 0.218866000 |
| H | 2.955148000 | 0.570287000 | 2.027332000 |
| H | 3.494181000 | -0.952040000 | 1.658493000 |
| H | 1.608884000 | -3.270717000 | -0.442225000 |
| H | 2.727258000 | -2.044216000 | -0.184109000 |
| C | 1.017166000 | 0.454647000 | -1.347104000 |
| C | 0.847949000 | 1.548921000 | -0.580636000 |
| C | -0.493904000 | 1.893039000 | 0.003755000 |
| C | -1.626182000 | 0.960769000 | -0.287688000 |
| C | -1.451459000 | -0.136885000 | -1.051700000 |
| C | -0.120306000 | -0.486169000 | -1.626878000 |
| C | 2.286317000 | 0.088651000 | -1.895616000 |
| C | 1.927162000 | 2.425770000 | -0.247347000 |
| N | 3.310733000 | -0.222327000 | -2.337733000 |
| N | 2.803325000 | 3.127741000 | 0.036851000 |
| Cl | -2.720134000 | -1.222207000 | -1.424975000 |
| Cl | -3.126458000 | 1.394203000 | 0.395951000 |
| O | 0.063056000 | -1.478138000 | -2.293321000 |
| O | -0.628543000 | 2.893377000 | 0.664968000 |
| H | -0.591152000 | -3.275147000 | 0.665600000 |
| H | -2.140847000 | -1.970079000 | 2.096553000 |
| H | 0.773324000 | 0.802946000 | 2.978024000 |

## The XYZ coordinates of the optimized geometry for AMFP-DDQ in solvent

| | | | |
|---|---|---|---|
| C | 0.152623000 | -1.915494000 | -1.474175000 |
| C | 0.789392000 | -2.962280000 | -0.824939000 |
| N | 2.036386000 | -2.876667000 | -0.345621000 |
| C | 2.682557000 | -1.725242000 | -0.518502000 |
| C | 2.148604000 | -0.602987000 | -1.163853000 |
| C | 0.828370000 | -0.704170000 | -1.657603000 |
| N | 2.908709000 | 0.538561000 | -1.384688000 |
| N | 0.210531000 | 0.404561000 | -2.210885000 |
| H | 2.428940000 | 1.428427000 | -1.368945000 |
| H | 3.776240000 | 0.584899000 | -0.867270000 |
| H | 0.801921000 | 1.026817000 | -2.748789000 |
| H | -0.653178000 | 0.216460000 | -2.703990000 |
| C | 0.222466000 | 1.768568000 | 0.526673000 |
| C | 0.675067000 | 0.693912000 | 1.211182000 |
| C | -0.177833000 | -0.517136000 | 1.418273000 |
| C | -1.529226000 | -0.501931000 | 0.780949000 |
| C | -1.969005000 | 0.562405000 | 0.078959000 |

| | | | |
|---|---|---|---|
| C | -1.149610000 | 1.801157000 | -0.067866000 |
| C | 1.013339000 | 2.948359000 | 0.350518000 |
| C | 1.974839000 | 0.674454000 | 1.810165000 |
| N | 1.663915000 | 3.894042000 | 0.193902000 |
| N | 3.033985000 | 0.686524000 | 2.278159000 |
| Cl | -3.503962000 | 0.605465000 | -0.678974000 |
| Cl | -2.470292000 | -1.910553000 | 1.022600000 |
| O | -1.574642000 | 2.807057000 | -0.590720000 |
| O | 0.219197000 | -1.445143000 | 2.085073000 |
| H | -0.858362000 | -2.038861000 | -1.850318000 |
| H | 0.274569000 | -3.906356000 | -0.676599000 |
| H | 3.691581000 | -1.667048000 | -0.115321000 |

**The XYZ coordinates of the optimized geometry for AMFP-TCNE in gas phase**

| | | | |
|---|---|---|---|
| C | 0.914242000 | 1.051385000 | -1.412555000 |
| C | 1.833316000 | 1.901093000 | -0.800810000 |
| N | 2.813994000 | 1.474755000 | -0.006770000 |
| C | 2.887169000 | 0.158358000 | 0.213755000 |
| C | 2.026540000 | -0.787848000 | -0.335830000 |
| C | 1.017537000 | -0.325085000 | -1.201428000 |
| N | 2.166010000 | -2.177716000 | -0.131582000 |
| N | 0.110657000 | -1.229199000 | -1.738379000 |
| H | 1.384357000 | -2.593013000 | 0.367088000 |
| H | 3.022959000 | -2.414271000 | 0.352217000 |
| H | 0.478964000 | -2.171074000 | -1.820064000 |
| H | -0.362705000 | -0.923581000 | -2.578036000 |
| C | -1.766148000 | -0.518820000 | 0.414911000 |
| C | -1.289229000 | 0.685556000 | 0.831329000 |
| C | -2.768184000 | -0.617518000 | -0.602874000 |
| N | -3.580566000 | -0.693111000 | -1.424956000 |
| C | -1.264716000 | -1.739689000 | 0.972125000 |
| N | -0.845725000 | -2.719563000 | 1.425515000 |
| C | -0.292889000 | 0.780881000 | 1.855678000 |
| N | 0.496712000 | 0.853321000 | 2.699828000 |
| C | -1.783809000 | 1.910832000 | 0.275685000 |
| N | -2.190550000 | 2.898916000 | -0.170796000 |
| H | 0.148291000 | 1.454193000 | -2.069769000 |
| H | 1.768825000 | 2.973929000 | -0.960797000 |
| H | 3.682606000 | -0.172873000 | 0.879417000 |

**The XYZ coordinates of the optimized geometry for AMFP-TCNE in solvent**

| | | | |
|---|---|---|---|
| C | 0.940446000 | 1.065300000 | -1.377062000 |
| C | 1.874041000 | 1.896842000 | -0.763831000 |
| N | 2.859632000 | 1.446295000 | 0.013203000 |
| C | 2.922501000 | 0.122930000 | 0.211151000 |
| C | 2.045709000 | -0.804958000 | -0.343253000 |
| C | 1.029762000 | -0.316709000 | -1.190737000 |
| N | 2.185928000 | -2.193020000 | -0.159698000 |

| | | | |
|---|---|---|---|
| N | 0.102438000 | -1.192139000 | -1.731553000 |
| H | 1.361731000 | -2.638061000 | 0.231965000 |
| H | 2.985361000 | -2.432284000 | 0.413754000 |
| H | 0.438062000 | -2.141148000 | -1.852475000 |
| H | -0.387151000 | -0.856898000 | -2.550903000 |
| C | -1.776273000 | -0.518159000 | 0.405320000 |
| C | -1.297755000 | 0.688603000 | 0.812061000 |
| C | -2.794574000 | -0.620547000 | -0.596500000 |
| N | -3.624564000 | -0.696275000 | -1.400114000 |
| C | -1.283265000 | -1.737011000 | 0.974862000 |
| N | -0.880141000 | -2.715647000 | 1.444177000 |
| C | -0.295357000 | 0.792745000 | 1.830353000 |
| N | 0.500491000 | 0.877758000 | 2.666936000 |
| C | -1.800519000 | 1.910951000 | 0.256723000 |
| N | -2.215145000 | 2.896036000 | -0.188137000 |
| H | 0.173273000 | 1.484769000 | -2.021527000 |
| H | 1.817493000 | 2.972030000 | -0.908568000 |
| H | 3.722467000 | -0.229392000 | 0.859518000 |

**The XYZ coordinates of the optimized geometry for AMFP in gas phase**

| | | | |
|---|---|---|---|
| C | -0.746887000 | 1.349851000 | 0.000000000 |
| C | -1.909256000 | 0.589042000 | 0.000000000 |
| N | -1.911096000 | -0.744361000 | 0.000000000 |
| C | -0.719740000 | -1.344159000 | 0.000000000 |
| C | 0.513243000 | -0.689736000 | 0.000000000 |
| C | 0.500454000 | 0.721274000 | 0.000000000 |
| N | 1.692893000 | -1.418403000 | 0.000000000 |
| N | 1.665246000 | 1.459533000 | 0.000000000 |
| H | 1.658457000 | -2.421856000 | 0.000001000 |
| H | 2.602656000 | -1.000722000 | -0.000001000 |
| H | 1.622802000 | 2.462756000 | 0.000001000 |
| H | 2.577929000 | 1.046886000 | 0.000002000 |
| H | -0.806746000 | 2.434489000 | 0.000000000 |
| H | -2.879896000 | 1.077362000 | 0.000000000 |
| H | -0.731380000 | -2.433932000 | 0.000000000 |

**The XYZ coordinates of the optimized geometry for TCNE in gas phase**

| | | | |
|---|---|---|---|
| C | 0.000000000 | 0.679992000 | 0.000000000 |
| C | 0.000000000 | -0.679992000 | 0.000000000 |
| C | 1.222881000 | 1.426681000 | 0.000000000 |
| N | 2.206113000 | 2.038453000 | 0.000000000 |
| C | -1.222881000 | 1.426681000 | 0.000000000 |
| N | -2.206113000 | 2.038453000 | 0.000000000 |
| C | -1.222881000 | -1.426681000 | 0.000000000 |
| N | -2.206113000 | -2.038453000 | 0.000000000 |
| C | 1.222881000 | -1.426681000 | 0.000000000 |

| | | | |
|---|---|---|---|
| N | 2.206113000 | -2.038453000 | 0.000000000 |

**The XYZ coordinates of the optimized geometry for DDQ in gas phase**

| | | | |
|---|---|---|---|
| C | 1.400694000 | 0.674600000 | 0.000000000 |
| C | 1.400694000 | -0.674600000 | 0.000000000 |
| C | 0.116749000 | -1.455772000 | 0.000000000 |
| C | -1.160155000 | -0.674414000 | 0.000000000 |
| C | -1.160155000 | 0.674414000 | 0.000000000 |
| C | 0.116749000 | 1.455772000 | 0.000000000 |
| C | 2.608889000 | 1.440279000 | 0.000000000 |
| C | 2.608889000 | -1.440279000 | 0.000000000 |
| N | 3.592585000 | 2.051201000 | 0.000000000 |
| N | 3.592585000 | -2.051201000 | 0.000000000 |
| Cl | -2.590314000 | 1.608447000 | 0.000000000 |
| Cl | -2.590314000 | -1.608447000 | 0.000000000 |
| O | 0.136272000 | 2.661940000 | 0.000001000 |
| O | 0.136272000 | -2.661940000 | 0.000001000 |
